# Bilateral corneal perforation and iris prolapse as a complication non-peripheral ulcerative keratitis in a patient with fulminant granulomatosis with polyangiitis: a case report

**DOI:** 10.1186/s12348-020-0195-6

**Published:** 2020-01-10

**Authors:** Andrés Vargas-Villanueva, Natalia Carvajal-Saiz, Juliana Muñoz-Ortiz, Alejandra de-la-Torre

**Affiliations:** 1 Escuela Superior de Oftalmología—Instituto Barraquer de América, Bogotá, Colombia; 2 NeURos Research Group, Escuela de Medicina y Ciencias de la Salud, Universidad del Rosario, Carrera 24 # 63, C 69 Bogotá, Colombia

## Introduction

Granulomatosis with polyangiitis (GPA) (formerly known as Wegener´s granulomatosis) is a systemic necrotizing vasculitis belonging to a heterogeneous group of systemic anti-neutrophil cytoplasmic antibody (ANCA) associated vasculitis which affects small and medium-sized blood vessels [1, 2]. The annual incidence of GPA has been estimated to be 8 to 10 million, has a peak age of onset of 64 to 75 years [3], and the frequency of presentation in female and male individuals is similar [4].

Constitutional signs like fever, asthenia, and weight loss are frequent (50%) but non-specific. Ear, nose, and throat signs (crusting rhinorrhea, sinusitis, chronic otitis, or damage of the facial cartilage) are present in 70 to 100% of cases at diagnosis [5]. Lung involvement, characterized by alveolar hemorrhage or parenchymatous nodules, affects 50 to 90% of patients [4]. The focal segmental necrotizing glomerulonephritis is the most frequent renal involvement in 40 to 100% of cases [6]. Involvement of the nervous system, the central nervous system, the pachymeninges, the heart, the pericardium, and the gastrointestinal system are less frequent, observed in a range of 5 to 40% of cases [1].

Ocular involvement is frequent, typically in the form of necrotizing nodular episcleritis, peripheral corneal ulcerations, and retinal vasculitis. The involvement of the eye socket is rare and can be characterized by a granulomatous retro-orbital pseudotumor or as dacryoadenitis. Inflammatory exophthalmia can also be a sinus inflammation manifestation [1].

We report a case of a male patient with GPA diagnosis with severe and fulminant progression of the disease and atypical ocular manifestations.

## Case presentation

A 58-year-old male patient presented at the emergency department with a 2-month history of ocular symptoms of conjunctival injection, pruritus, photophobia, and decreased vision in both eyes. Concomitantly, he referred a cough, hyaline rhinorrhea, subjective fever, weight loss, asthenia, adynamia, and edema of the lower limbs. In the last 15 days, he presented diuresis with macroscopic hematuria and 8 days of purulent discharge from the eyes. The only antecedents the patient referred were smoking for 20 years with an index of 20 packs/year and a chronic exposure to rice crop fumigation toxins.

**Fig. 1:**
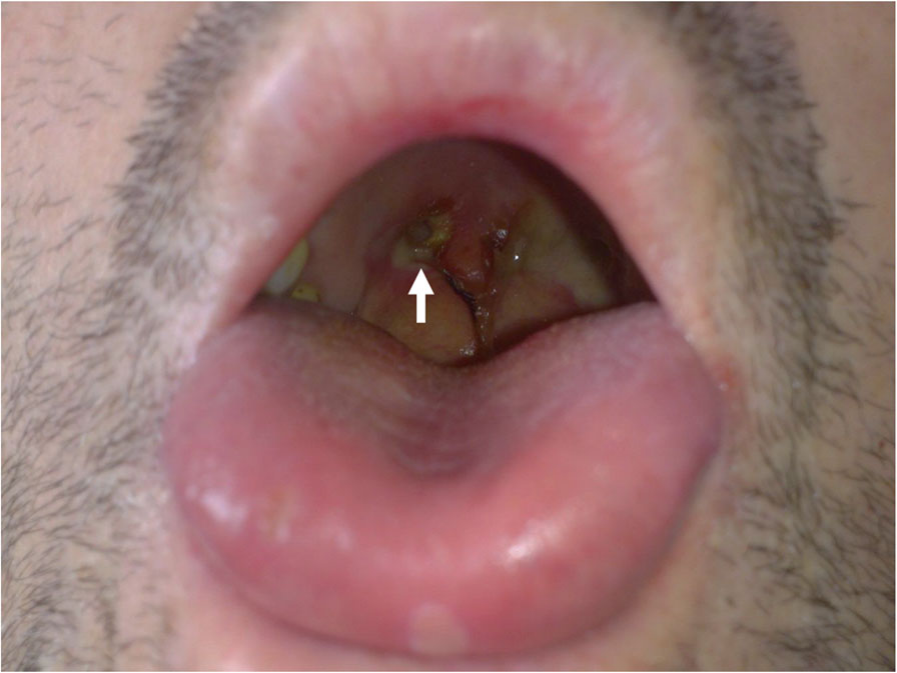
Nasopharyngeal involvement in GPA. The picture shows a mucosal ulcer with necrotic borders at the level of the joint between soft and hard palate (white arrow)

The physical examination revealed multiple painful hypo-pigmented lesions of 1 × 1 cm on the tongue and cheek mucosa, and a painless mucosal ulcer with necrotic borders at the level of the joint between the soft and hard palate of approximately 4 cm in diameter (Fig. 1). Pulmonary auscultation revealed bilateral basal hypoventilation and dullness in the percussion. The joints evaluation was normal.

**Fig. 2:**
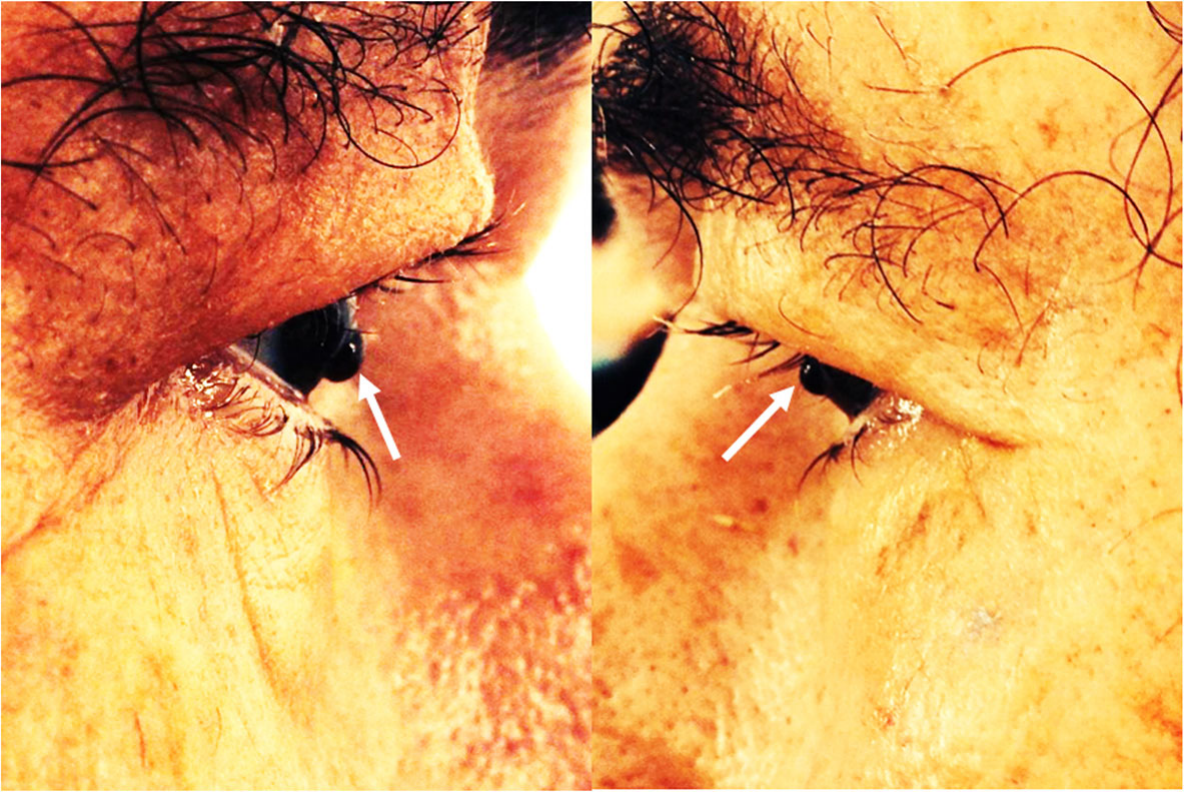
Ocular involvement in GPA. Iridian herniation through the perforation of the corneas (white arrows)

**Fig. 3:**
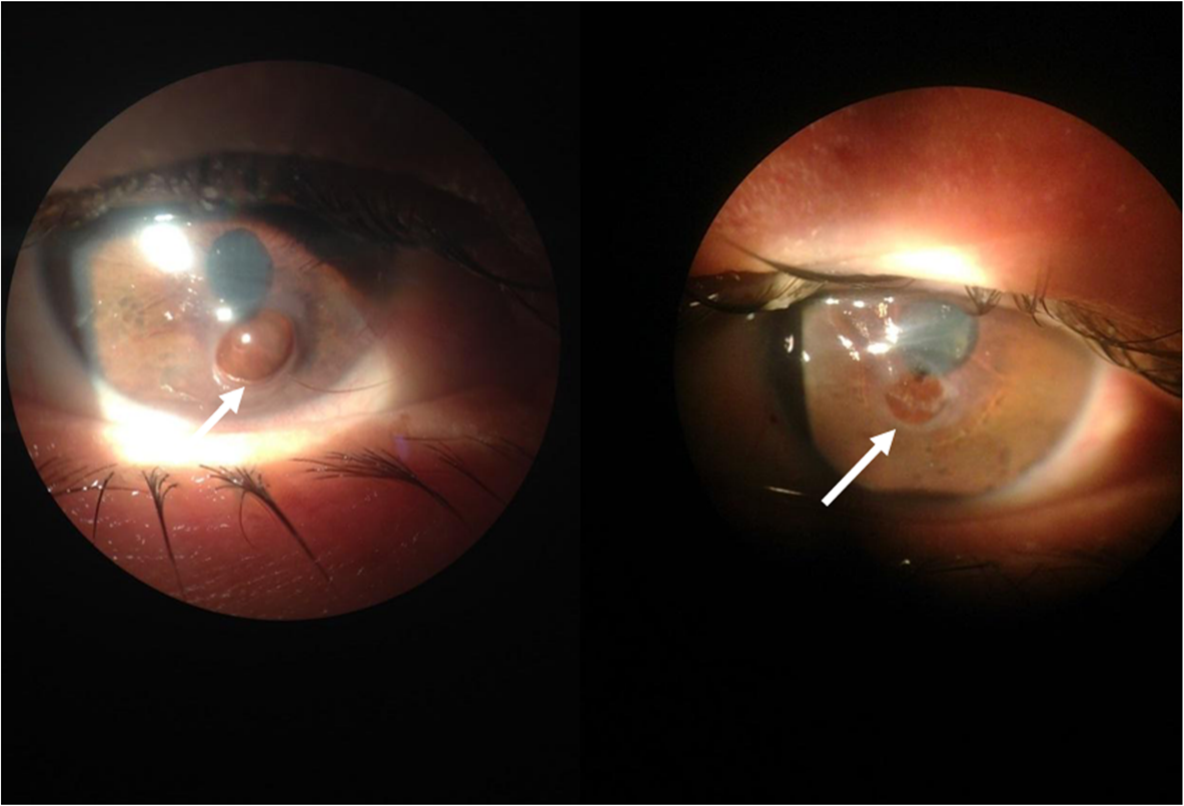
Ocular involvement in GPA. Bilateral corneal ulcers, in OD inferior-paracentral located (white arrow) with iris exposure and in OS centrally located (white arrow) with iris exposure, mild corneal edema, and athalamia

The initial ophthalmologic evaluation revealed visual acuity of hand movement in OU, and external examination allowed the evaluation of iridian herniation through the corneal melting (Fig. 2). Biomicroscopy assessment showed bilateral corneal ulcers, in OD inferior-paracentral located with iris exposure and in OS centrally located with iris exposure, mild corneal edema and athalamia (Fig. 3), non-reactive pupils, and nuclear lens sclerosis in both eyes. Intraocular pressure (IOP) was not measured due to the presence of corneal perforation and indirect ophthalmoscopy evaluation was not possible due to the opacity of the medium. Physical examination yielded no other positive findings.

**Fig. 4:**
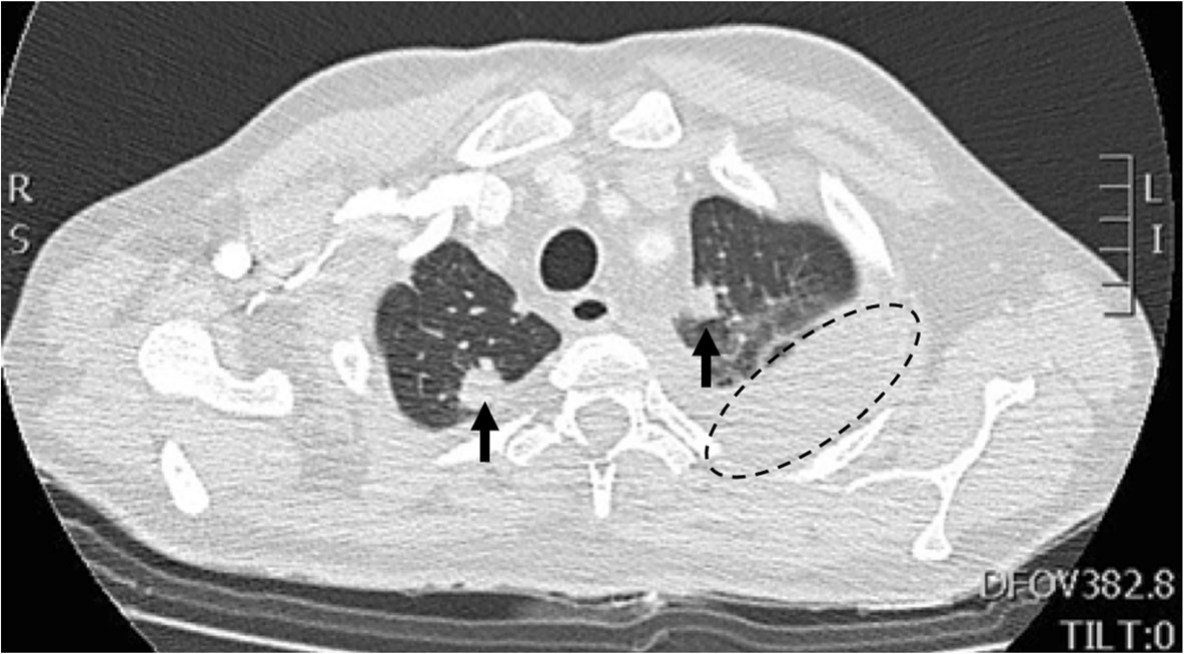
Pulmonary involvement in GPA. Bilateral hyperdense pulmonary nodules (black arrows) located in both lung apices with bilateral pleural effusion (black circle), associated with pericardial effusion and pre-capillary pulmonary hypertension

**Fig. 5:**
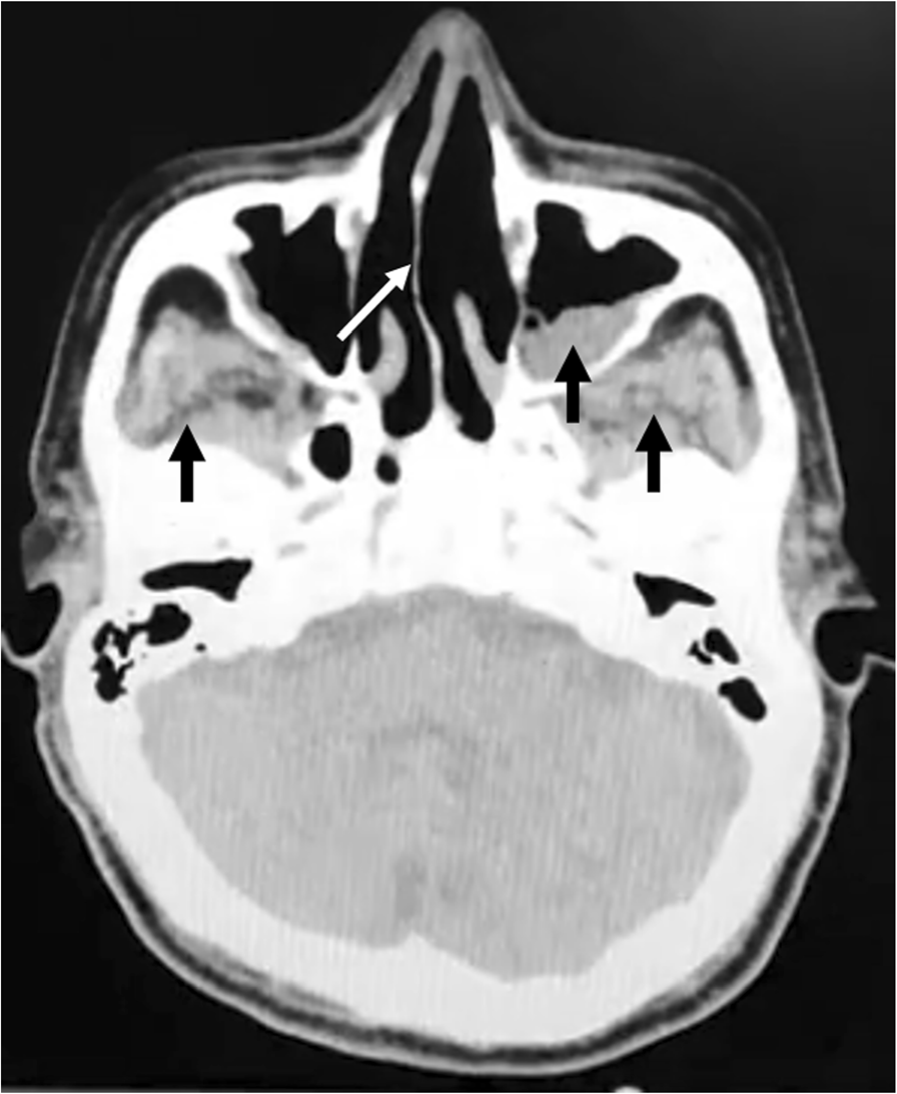
Paranasal sinuses CT. Pansinusitis (black arrows) with a right deviation of the nasal septum (white arrow)

Thoracic high resolution computed tomography (HR-CT) was performed, showing bilateral hyperdense pulmonary nodules located in both lung apices with bilateral pleural effusion, associated with pericardial effusion and pre-capillary pulmonary hypertension (Fig. 4). Paranasal sinuses CT showed acute pansinusitis with a right deviation of the nasal septum (Fig. 5). The urinalysis revealed hematuria, proteinuria in 24-h urine collection and hypoalbuminemia.

With the previously described findings, neoplasia was initially suspected, and so specific management was not immediately established. After several days, the immunological profile showed positive c-anti-neutrophil cytoplasmic antibody (c ANCA). Given the findings to the physical examination, the laboratory tests and images, the GPA was diagnosed according to the ACR criteria of 1990 [7] (sensitivity 88.3%, specificity 92%) fulfilling three of the four possible criteria:

1. *Clinical criteria. Painful or painless ulcers in the oral mucosa or purulent nasal or blood flow*
2. *Biological criteria. Micro hematuria > 5 red blood cells or presence of blood cylinders*
3. *Radiological criteria. Infiltrates, nodules, or pulmonary cavities not migratory or fleeting*
4. Histological criteria. Granulomatous inflammation within the wall of an artery, peri-vascular, or extra-vascular of an artery or arteriole

After diagnosis, the patient received treatment with 1 g of methylprednisolone per day for 3 days and two doses of cyclophosphamide were administered according to the EUVAS protocol [8]. Eye treatment was administered with permanent bilateral occlusion, quinolone, topical steroids and carboxymethylcellulose q4h. Subsequently, visual acuity improved slightly to 20/200 in the right eye and 20/400 in the left eye, while the mucosa of the palate was completely healed and the radiographic pulmonary pattern decreased in subsequent control scans. Despite immunosuppressive therapy and clinical improvement, the patient died after 2 months of treatment due to ventilatory failure secondary to multilobar pneumonia.

## Discussion

### Definition

Granulomatosis with polyangiitis, or formerly known as Wegener’s granulomatosis (WG), is an inflammatory disease first described in 1931 by Heintz Klinger as a variant of polyarteritis nodosa, and then in greater detail as a separate syndrome by Wegener in 1936–1939 [9]. GPA is an autoimmune small-vessel vasculitis characterized by systemic necrotizing vasculitis, necrotizing granulomatous inflammation, and necrotizing glomerulonephritis, highly associated with anti-neutrophil cytoplasmic antibodies (ANCA) [10].

### Etiology

The etiology of GPA is not well defined; however, a strong association has been found with various epigenetic factors. This includes infections (bacterial, mycobacterial, fungal, or viral) and the nasal transport of *Staphylococcus aureus* is a common trigger of GPA outbreaks, environmental (contamination, smoking, inhaled toxins, inhaled chemicals, and exposure to metals), and drugs (antibiotics such as cefotaxime, antithyroid drugs such as benzylthiouracil, anti-tumor necrosis factor-alpha agents such as adalimumab, psychoactive drugs such as clozapine, and other drugs such as allopurinol, cocaine, among others). Susceptibility to proteinase-3 associated with ANCA is an associated genetic factor [10]. Our patient had a 20-year smoking history and chronic exposure to toxins from rice crop fumigants—factors that make us think of probable etiologies triggering his disease.

### Clinical spectrum

Clinical manifestations are diverse, including constitutional symptoms such as general malaise, myalgia, arthralgia, anorexia, weight loss, and pyrexia [10], some of which were present in our patient. Granulomatous and necrotizing inflammatory lesions in the upper and lower respiratory tracts are common in GPA patients, and they are part of the diagnostic criteria of the disease; in the same way, renal diffuse pauci-immune glomerulonephritis is usual in these patients and may be a rapidly progressive syndrome [1]. The typical laboratory findings include hematuria, proteinuria, and cellular casts on urine cytology. Dermatologic signs are leucocytoclastic vasculitis, digital infarcts, purpura, cutaneous ulcers, and gangrene, although these are non-specific manifestations [10].

Involvement of the eye, central nervous system, pachymeninges, the heart, the pericardium, and the gastrointestinal system are less frequently [1].

### Epidemiology

GPA has an annual incidence of 8 to 10 million, with a peak age of onset of 64 to 75 years [3]. It is usually common in the white population [11] and men and women are affected with similar frequency [1].

### Ocular involvement

Ocular complications have been described in patients with GPA and may be the sole clinical presentation prior to systemic symptoms [12]. Such complications occur fairly frequently in 14 to 60% of cases, often in the form of necrotizing nodular episcleritis, scleritis, peripheral corneal ulceration, and retinal vasculitis. It is frequently bilateral, affecting14–30% of cases [13] and can cause significant and often irreversible morbidity in 8–17% of cases if not treated appropriately [11].

The ocular manifestations can be categorized according to the structures involved. The orbit compromise is usually attributed to contiguous spread from the adjacent sinuses [14], and some of the most commonly encountered manifestations are proptosis, diplopia, and decreased vision, secondary to mass compression with nerve involvement (ischemic optic neuropathy) and entrapment of extraocular muscle [4, 15]. Eyelid changes in GPA may include edema, entropion, trichiasis, and xanthelasma [16]. Dacryoadenitis has been reported as a sign of GPA, secondary to nasolacrimal duct obstruction, a late finding associated with nasal involvement of this disease [17]. Conjunctival involvement includes chronic inflammation, granuloma formation, or ulceration, as well as biopsy findings that can contribute to the diagnosis of the disease [18].

The uveitis associated with GPA is non-specific. In the case of anterior uveitis, it usually coexists with scleritis, a fact suggesting that uveitis may be a secondary phenomenon [19]. Retinal and choroidal involvement is uncommon. However, bilateral arterial and vein occlusions of the retinal and choroidal circulations, vitreous hemorrhage, retinal hemorrhages, retinal edema, cotton wool exudates, and choroidal thickening have all been previously reported [13]. Our patient did not present any of the previously described manifestations; however, the severe course of the disease did not allow for long-term follow-up, where there is a possibility that he may have had ocular comorbidities other than peripheral ulcerative keratitis (PUK).

PUK is the most significant corneal complication of GPA. In the medical literature, two hypotheses of the physiopathology of this manifestation have been described. The first, based on the histopathological findings, expose an immune-mediated occlusive necrotizing vasculitis of the anterior ciliary arteries that supply the anterior segment of the eye, leading to ulceration of the peripheral cornea and often accompanied by scleritis (usually necrotizing) [13]. The second refers to the absence of other early signs of anterior segment ischemia, and the early presence of marginal infiltrates that may be an indication of the immunological deficit suffered by patients with GPA, as well as their failure to overcome a bacterial infection that may be an initiating or stimulatory factor for the vasculitis and subsequent tissue necrosis [14]. In both cases, reference is made to the peripheral corneal involvement, which is why the case of our patient is considered unusual, given the very well demarcated paracentral involvement of the cornea with healthy epithelium around it on his OS.

### Treatment

The GPA *Remission-Induction Therapy* combines glucocorticoids (GC) and cyclophosphamide (CYP), a scheme that has repeatedly been demonstrated to achieve remission in the majority of patients [20]. CYP can be discontinued when clinical remission is obtained, usually, after 3–6 months, and GC, after a high initial dose of 1 mg/kg/day of prednisone-equivalent, should be tapered to 5 mg/day, this maintenance dose can be continued 6 or 12 months after the initial disease flare [21]. For maintenance therapy, less aggressive treatments are used, e.g., Methotrexate and Azathioprine (combined with low-dose GC), although favorable responses have been reported with Mycophenolate mofetil, Leflunomide, and cyclosporine in small trials [22].

Ocular involvement does not readily respond to topical agents, except in some cases of episcleritis, conjunctivitis, and anterior uveitis, where a short course of topical steroids can be beneficial. Treatment of keratitis in systemic vasculitis is largely aimed at treating the underlying disorder. In cases of imminent perforation in peripheral ulcerative keratitis, local measures such as adhesive glue or graft may be necessary. In cases of intermediate uveitis, subconjunctival steroid injections may be required, with oral prednisone reserved for chronic posterior uveitis or refractory cases [11].

The relationship between ocular symptoms and the progression of the disease or mortality of patients with GPA has not yet been established. In a case series of 8 patients with GPA and ocular compromise, reported by Spalton et al. in 1981, 3 of the 8 presented PUK of approximately 4 months evolution prior to diagnosis. In all 8 cases, after the initiation of immunosuppressive treatment with corticosteroids or in combination therapy with azathioprine, the patients demonstrated a quiescence of the disease in follow-up periods ranging from 1 to 8 years [14]. In comparison, our patient presented a severe disease phenotype that began with ocular symptomatology and suffered a rapid progression of the disease that led to death in a period of 2 months following the onset of symptoms despite immunosuppressive management.

## Conclusion

Here, we present a case of a patient with GPA, in his debut course with atypical ocular manifestations given by bilateral ulcerative keratitis with central involvement in OS. It is important to consider this type of presentation since diagnosis and establishment of its treatment were delayed as a result of not suspecting this entity at an earlier stage. Early diagnosis and an appropriate interdisciplinary approach are required to decrease recurrence, as well as morbidity and mortality, in patients with GPA-mediated inflammatory ocular disease.

## Data Availability

All data generated or analyzed during this study are included in this published article and available from the corresponding author on reasonable request.

## Abbreviations

ACR: American College of Rheumatology
ANCA: Anti-neutrophil cytoplasmic antibody
CT: Computed tomography
CYP: Cyclophosphamide
EUVAS: European Vasculitis Society
GC: Glucocorticoids
GPA: Granulomatosis with polyangiitis
HR-CT: Thoracic high resolution computed tomography
IOP: Intraocular pressure
OD: Right eye
OS: Left eye
PUK: Peripheral ulcerative keratitis
WG: Wegener´s granulomatosis
